# Immigrant, racialised and ethno-culturally diverse communities and community treatment orders: A scoping review

**DOI:** 10.1177/00207640251353689

**Published:** 2025-08-31

**Authors:** Hira Ahmad, Alasdair Forrest, Martin Rotenberg

**Affiliations:** 1 University of Aberdeen, UK; 2 Royal Cornhill Hospital, NHS Grampian, Aberdeen, UK; 3 Centre for Addiction and Mental Health, Toronto, ON, Canada; 4 Department of Psychiatry, University of Toronto, ON, Canada

**Keywords:** Ethnicity, immigrant, racialised groups, community treatment order, scoping review

## Abstract

**Background:**

People with serious mental illness from immigrant, racialised and ethno-culturally diverse communities experience greater coercion in mental health care.

**Aims:**

This review aims to scope the literature and synthesise findings relevant on the association between these groups, and the use of Community Treatment Orders (CTOs) and related forms of compulsory community treatment.

**Method:**

Five electronic databases were searched to identify relevant studies. Studies were included if they directly examined the association between immigrant, racialised and ethno-culturally diverse communities and community treatment orders and related forms of treatment or presented relevant data as part of a larger study.

**Results:**

Of the 43 studies included 9 studies focussed directly on associations between immigrant, racialised and ethno-culturally diverse communities and CTOs, while 34 studies presented relevant data related to immigrant, racialised and ethno-culturally diverse communities and CTOs albeit not the primary focus of the study. Most studies were quantitative, with varied study designs. The majority of studies were from Australia and New Zealand. followed by North America and the UK. Of the nine studies focussing directly on ethnicity and CTOs, results were mixed, and varied based on design, population and jurisdiction.

**Conclusions:**

The relationship between ethnicity, immigration status and CTOs is complex, with mixed findings across jurisdictions. Most of the literature comes from Australia and New Zealand, were Indigenous populations were a significant focus. The review highlights the need for more qualitative and quantitative research, especially in underrepresented ethnic groups and jurisdictions outside of Australia and New Zealand.

## Background

Community Treatment Orders (CTO) are legal frameworks that compel a person with a mental disorder to take treatment and engage with services. CTOs and other related compulsory community treatment frameworks (i.e. Outpatient Commitment [OPC], Assisted Outpatient Treatment [AOT], Mandared Community Treatment), exist in many jurisdictions across the world such as Australia, New Zealand, the United States, Asia, Canada, United Kingdom and Switzerland (Mikellides et al., 2019). CTOs were introduced in England and Wales in 2007 as part of the Mental Health Act (Care Quality Commission, 2022) and introduced in Scotland in 2005 (Lawton-Smith, 2006) to reduce the risk of readmission to hospital of patients who typically have frequent attendances to hospital and to facilitate improved adherence to treatment post-discharge (Burns & Molodynski, 2014). Additional benefits may include facilitating access to community psychiatric care teams and supporting greater family involvement (Beaglehole & Tennant, 2023).

Generally, a CTO may be issued if there are significant concerns about the patient’s safety or the safety of others. CTOs are initiated by clinicians in the United Kingdom, Australia and New Zealand, but are ordered by the court in the United States (Gray et al., 2016). Additionally, there is often a requirement for the patient to lack the capacity to consent to the treatment plan for a CTO to be implemented (Beaglehole & Tennant, 2023). In some jurisdictions, a CTO can also be issued due to the risk of substantial mental or physical deterioration, which sets a lower threshold than the risk of harm to self and others. In addition to this, further criteria must also be met depending on the jurisdiction, including the duration of hospital stay, the number of hospitalisations, the patient’s response to treatment and obtaining consent from a substitute decision-maker (Gray et al., 2016).

Despite the potential benefits of reducing hospitalisations and maintaining people in community settings, concerns have also been raised that CTOs have not reduced readmission to hospital but rather have restricted patients’ freedoms (Burns et al., 2013). There are mixed findings in this regard and it is important to highlight the challenging nature of studying these forms of treatment in randomised controlled trials due to the ethics and feasibility of these trials (Burns et al., 2013). In addition, most studies often use observational data, which can bias findings and limit generalisability to other settings (Song & Chung, 2010). Nuance is required to interpret CTO-associated outcomes as a variety of factors can impact outcomes. For example, studies have shown that CTOs, issued after a first admission, were linked to a higher risk of readmission, whereas their use after subsequent admissions was linked with a reduced risk of readmission (Burgess et al., 2006). Considering the increasing use of CTOs across different jurisdictions globally (Kisely et al., 2004; Light et al., 2012; NHS Digital, 2022), it is important to understand how factors which have found to be associated with other coercive treatments and negative care pathways are associated with CTO placement and outcomes (Bardell-Williams et al., 2019; Bhui et al., 2003).

People from racialised communities experience more negative pathways to care and coercion during care. People from these communities are more likely to experience coercive treatment (Bansal et al., 2022; Bhui et al., 2003), are more likely to be detained for involuntary admissions (Barnett et al., 2019), and may be exposed to greater levels of stigma (Eylem et al., 2020). In addition, there is a long-standing history of increased risk of serious mental illness in these populations (Bourque et al., 2011), and they are also less likely to receive potentially beneficial evidence-based mental health support (NHS Digital, 2016). There have been calls for a change in UK legislation to address the level of coercion and disparities in treatment in diverse and racialised communities (Garratt, 2024).

CTO placement can vary depending on different sociodemographic characteristics, including age, sex, education, ethnic origin and substance misuse, among others (Lei et al., 2019). Recent NHS data from 2020 to 2023 highlighted that CTO use in Black or Black British people were over 8 to 10 times higher than for White people (NHS Digital, 2020, 2021, 2022, 2023). There is also international literature highlighting no differences in CTO use in the USA among African Americans when compared to others (Galon et al., 2012), and mixed findings with respect to CTO use in indigenous communities in New Zealand (Newton-Howes et al., 2014).

Examining potential disparities in CTO usage across different ethnic groups should be a critical focus in mental health research, particularly in considering higher rates of serious mental illness in these communities (Bourque et al., 2011), differences in accessing evidence-based care and treatment, as well as the potential impact of coercive care on ongoing engagement and treatment for people who are experiencing chronic mental health issues (Bansal et al., 2022; Bhui et al., 2003; Morgan et al., 2004).

In this review, we use the terms ‘ethnicity’, ‘immigrant’ and ‘racialised groups’ to reflect the varying ways that social and cultural identity is framed across jurisdictions and studies. ‘Ethnicity’ refers to shared cultural practices, language and ancestry. ‘Immigrant populations’ are defined as individuals born outside the country of study, typically defined based on place of birth or migration status. ‘Racialised groups’ refers to populations that are categorised and treated differently based on perceived race, often in ways that are socially and institutionally constructed rather than biologically determined. In some studies, overlapping or jurisdiction-specific terms such as ‘culturally and linguistically diverse (CALD)’ is also used. These definitions vary across jurisdictions and studies, and care has been taken to interpret results within each study’s specific context.

While prior reviews provide regional insights (Kisely, 2016; Kisely et al., 2004, 2015) there are currently no reviews which focus specifically on ethnicity, immigrant and racialised groups in relation to CTOs on a global scale. Therefore, given the growing body of literature on these topics, a scoping review is warranted to synthesise the current research on the relationship between ethnicity and CTOs, map the literature and identify gaps and areas that warrant further study and attention.

## Methods

This scoping review uses Arksey and O’Malley’s scoping review methodology (Arksey & O’Malley, 2005), further developed by Levac et al. (2010). This approach is intended to map the current state of knowledge and identify gaps in the literature. Unlike systematic reviews, the research questions in this type of review are broader and it does not require a risk of bias assessment of included studies (Munn et al., 2018). An attempt was made to follow the reporting guidelines outlined in the PRISMA extension for scoping reviews (PRISMA-ScR; Tricco et al., 2018).

### Search strategy

**Figure A1.**
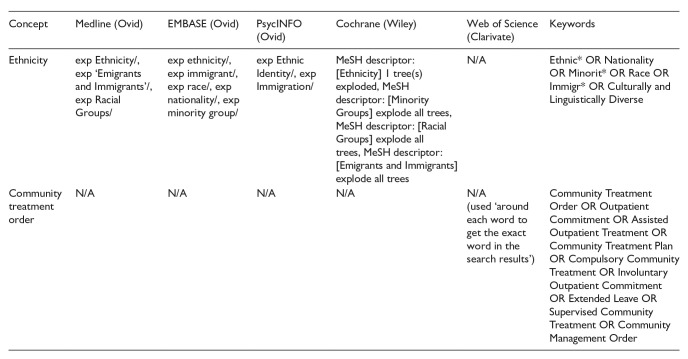
Search terms.

A structured literature search was conducted by the primary author using MEDLINE, Embase, PsycINFO, Cochrane Library and Web of Science (Tricco et al., 2018) up until July 24, 2024, with no limitations on the date range or language of papers screened. A Google Advanced search was also conducted with an a priori decision to search the first 10 pages of Google Advanced to ensure a substantive review from that database. The search strategy included a combination of (Ethnic* OR Nationality OR Minorit* OR Race OR Immigr* OR Culturally and Linguistically Diverse) AND (Community Treatment Order OR Outpatient Commitment OR Assisted Outpatient Treatment OR Community Treatment Plan OR Compulsory Community Treatment OR Involuntary Outpatient Commitment OR Extended Leave OR Supervised Community Treatment OR Community Management Order). The search strategy was adapted for each database, informed by an initial pilot search and reviewed by a second researcher. Keyword searching and controlled vocabulary were used where relevant, and the full search strategy is available in Appendix A (Figure A1). If terminology like ‘sociodemographic factors’ or ‘patient characteristics’ were mentioned in the title or abstract during the screening process, this was deemed sufficient for a full text review to clarify whether ethnicity or related concepts were present in the study.

Reference lists of included studies were reviewed for additional papers, and forward/backward reference searching was performed using citation chaser (Haddaway et al., 2021).

### Inclusion and exclusion criteria

Reviewed articles were screened against the following inclusion criteria: 1. The study included concepts related to ethnicity, racialised groups, immigrant status, 2. The study focussed on CTOs or related frameworks, for example, Outpatient Commitment or Supervised Community Treatment etc. and 3. The study was a qualitative, quantitative, mixed-methods study or a review of such studies. We excluded articles that were opinion or perspective pieces or were not peer-reviewed.

### Screening

Titles and abstracts were screened by the primary author (HA) against clear pre-specified inclusion/exclusion criteria, following a pilot screening phase and in conjunction with regular team meetings. Considering scoping review frameworks (Arksey & O’Malley, 2005; Levac et al., 2010) don’t explicitly mandate dual screening we opted to move forward with a single screener and have highlighted limitations in the relevant ‘Discussion’ section: ‘Limitations to the current review’.

### Full-text review and data extraction

The primary author (HA) extracted relevant data from included studies following a review of full-text screened articles against the inclusion and exclusion criteria by both the primary author and a second reviewer (MR). Reviewers disagreed on three full-text articles, resulting in a percentage agreement of 93.5%. Any discrepancies or conflicting opinions raised by the reviewers were addressed through discussion and consensus building between the reviewers and involvement of a third reviewer (AF) if consensus could not be formed. A second team member reviewed a random sample (25%) of extractions to verify accuracy. Limitations of this approach are reviewed in the relevant ‘Discussion’ section: ‘Limitations to the current review’.

The data extraction included: author, year and country of the publication, study objectives, study design, form of compulsory/mandated community treatment the study focussed on, population/sample, setting, data sources/measures, Immigrant, Ethnicity or Racialised group and relevant findings. We summarised the included studies and presented a descriptive synthesis of key findings (Appendix B – Table B1).

## Results

A total of 2,487 records were retrieved for review, with 92 duplicates removed, resulting in 2,395 articles for title and abstract screening. Following this, 2,226 records were excluded, leaving 169 full-text articles for eligibility assessment. Of these, 29 studies met inclusion criteria.

Through forward and backward reference searching, 14 additional studies were found and included for full-text review.

A total of 43 studies were included in the final review (Appendix A – Figure A2).

### Study characteristics

Appendix B (Table B1) presents a summary of the included literature. Among the 43 included studies, 9 had a primary focussed on the relationship between CTOs and ethnicity, immigration or racialised backgrounds (Galon et al., 2012; Gibbs et al., 2004; Kisely et al., 2020, 2021; Kisely & Xiao, 2018; Mfoafo-M’Carthy, 2014; Moss et al., 2019; Newton-Howes et al., 2014; Swanson et al., 2009), while the other 34 studies did not have a primary focus on this relationship but presented relevant data (Bardell-Williams et al., 2019; Barkhuizen et al., 2020; Beaglehole et al., 2021, 2023; Brophy et al., 2006; Burgess et al., 2006; Dey et al., 2021; Evans et al., 2010; Gonzales et al., 2015; Kisely, 2016; Kisely et al., 2004, 2017, 2020; Kisely, Preston, Xiao, Lawrence, Louise, & Crowe, 2013; Kisely, Preston, Xiao, Lawrence, Louise, Crowe, & Segal, 2013; Kisely, Xiao, Crowe, et al., 2014; Kisely, Xiao, Lawrence, & Jian, 2014; Lambert et al., 2009; Monnery & Belgamwar, 2011; Morandi et al., 2017; O’Brien et al., 2009; Ogilvie & Kisely, 2022; Paduret & Kelbrick, 2023; Parker et al., 2021; Patel et al., 2011; Segal & Burgess, 2006; Segal et al., 2017, 2009; Silva et al., 2019; Suetani et al., 2022; Swartz et al., 2001, 2002; Van Dorn et al., 2006; Weich et al., 2020).

Most studies were quantitative (Bardell-Williams et al., 2019; Barkhuizen et al., 2020; Brophy et al., 2006; Burgess et al., 2006; Beaglehole et al., 2021, 2023; Dey et al., 2021; Evans et al., 2010; Galon et al., 2012; Gonzales et al., 2015; Kisely, 2016; Kisely et al., 2004, 2015, 2020, 2021, 2023; Kisely, Preston, Xiao, Lawrence, Louise, & Crowe, 2013; Kisely, Preston, Xiao, Lawrence, Louise, Crowe, & Segal, 2013; Kisely & Xiao, 2018; Kisely, Xiao, Crowe, et al., 2014; Kisely, Xiao, Lawrence, & Jian, 2014; Lambert et al., 2009; Monnery & Belgamwar, 2011; Morandi et al., 2017; Moss et al., 2019; O’Brien et al., 2009; Ogilvie & Kisely, 2022; Paduret & Kelbrick, 2023; Parker et al., 2021; Patel et al., 2011; Segal & Burgess, 2006; Segal et al., 2009, 2017; Silva et al., 2019; Suetani et al., 2022; Swartz et al., 2001, 2002; Swanson et al., 2009; Van Dorn et al., 2006; Weich et al., 2020), with three being qualitative studies (Gibbs et al., 2004; Mfoafo-M’Carthy, 2014; Newton-Howes et al., 2014). There were two randomised controlled trials (Brophy et al., 2006; Ogilvie & Kisely, 2022), three systematic reviews (Kisely, 2016; Kisely et al., 2021, 2023) and 32 observational studies, predominantly cohort studies (Bardell-Williams et al., 2019; Barkhuizen et al., 2020; Beaglehole et al., 2021, 2023; Burgess et al., 2006; Dey et al., 2021; Kisely et al., 2004; Kisely et al., 2015, 2020; Kisely, Preston, Xiao, Lawrence, Louise, & Crowe, 2013; Kisely, Preston, Xiao, Lawrence, Louise, Crowe, & Segal, 2013; Kisely & Xiao, 2018; Kisely, Xiao, Crowe, et al., 2014; Kisely, Xiao, Lawrence, & Jian, 2014; Lambert et al., 2009; Monnery & Belgamwar, 2011; Morandi et al., 2017; Moss et al., 2019; O’Brien et al., 2009; Parker et al., 2021; Patel et al., 2011; Segal & Burgess, 2006; Segal et al., 2009, 2017; Silva et al., 2019; Swartz et al., 2001, 2002; Weich et al., 2020), alongside cross-sectional (Gonzales et al., 2015; Suetani et al., 2022; Swanson et al., 2009; Van Dorn et al., 2006) and miscellaneous designs (Evans et al., 2010; Galon et al., 2012; Paduret & Kelbrick, 2023). Of the qualitative studies, the majority used observational designs (Gibbs et al., 2004; Mfoafo-M’Carthy, 2014; Newton-Howes et al., 2014).

### Study location and publication year

Geographically, 20 studies were conducted solely in Australia (Bardell-Williams et al., 2019; Brophy et al., 2006; Burgess et al., 2006; Kisely et al., 2004, 2015, 2020; Kisely, Preston, Xiao, Lawrence, Louise, & Crowe, 2013; Kisely, Preston, Xiao, Lawrence, Louise, Crowe, & Segal, 2013; Kisely & Xiao, 2018; Kisely, Xiao, Crowe, et al., 2014; Kisely, Xiao, Lawrence, & Jian, 2014, Lambert et al., 2009; Morandi et al., 2017; Moss et al., 2019; Ogilvie & Kisely, 2022; Parker et al., 2021; Segal et al., 2009, 2017; Segal & Burgess, 2006; Suetani et al., 2022), 5 in New Zealand (Beaglehole et al., 2021, 2023; Dey et al., 2021; Gibbs et al., 2004; Newton-Howes et al., 2014), 5 in both Australia and New Zealand (Kisely et al., 2021, 2023), 6 in the USA (Galon et al., 2012; Gonzales et al., 2015; Swanson et al., 2009; Swartz et al., 2001, 2002; Van Dorn et al., 2006), 3 in Canada (Kisely, 2016; Mfoafo-M’Carthy, 2014; O’Brien et al., 2009), 6 in the UK (Barkhuizen et al., 2020; Evans et al., 2010; Monnery & Belgamwar, 2011; Paduret & Kelbrick, 2023; Patel et al., 2011; Weich et al., 2020) and 1 in Switzerland (Silva et al., 2019). Publication years ranged from 2001 to 2023, with the majority (72%) published in the last 15 years (Bardell-Williams et al., 2019; Barkhuizen et al., 2020; Beaglehole et al., 2021, 2023; Dey et al., 2021; Evans et al., 2010; Galon et al., 2012; Gonzales et al., 2015; Kisely, 2016; Kisely et al., 2015, 2020, 2021, 2023; Kisely, Preston, Xiao, Lawrence, Louise, & Crowe, 2013; Kisely, Preston, Xiao, Lawrence, Louise, Crowe, & Segal, 2013; Kisely & Xiao, 2018; Kisely, Xiao, Crowe, et al., 2014; Kisely, Xiao, Lawrence, & Jian, 2014; Mfoafo-M’Carthy, 2014; Monnery & Belgamwar, 2011; Morandi et al., 2017; Moss et al., 2019; Newton-Howes et al., 2014; Ogilvie & Kisely, 2022; Paduret & Kelbrick, 2023; Parker et al., 2021; Patel et al., 2011; Segal et al., 2017; Silva et al., 2019; Suetani et al., 2022; Weich et al., 2020).

### Types of mandated treatment

Community Treatment Order (CTO) were examined in 34 of the studies (Bardell-Williams et al., 2019; Barkhuizen et al., 2020; Beaglehole et al., 2021, 2023; Brophy et al., 2006; Burgess et al., 2006; Dey et al., 2021; Gibbs et al., 2004; Kisely, 2016; Kisely et al., 2004, 2015, 2020, 2021, 2023; Kisely, Preston, Xiao, Lawrence, Louise, & Crowe, 2013; Kisely, Preston, Xiao, Lawrence, Louise, Crowe, & Segal, 2013; Kisely & Xiao, 2018; Kisely, Xiao, Crowe, et al., 2014; Kisely, Xiao, Lawrence, & Jian, 2014; Lambert et al., 2009; Mfoafo-M’Carthy, 2014; Morandi et al., 2017; Moss et al., 2019; Newton-Howes et al., 2014; O’Brien et al., 2009; Ogilvie & Kisely, 2022; Paduret & Kelbrick, 2023; Parker et al., 2021; Patel et al., 2011; Segal et al., 2009, 2017; Silva et al., 2019; Suetani et al., 2022; Weich et al., 2020).

Other studies examined related treatment frameworks, for example, Outpatient Commitment (Galon et al., 2012), Compulsory Community Treatment (Beaglehole et al., 2021, 2023); Involuntary Outpatient Commitment (Swanson et al., 2009), Supervised Community Treatment, (Evans et al., 2010; Monnery & Belgamwar, 2011), Conditional Release (Segal & Burgess, 2006) and Mandated Community Treatment (Van Dorn et al., 2006), Assisted Outpatient Treatment (Gonzales et al., 2015). Forensic Orders were also examined separately as part of two studies, in addition to CTOs (Kisely et al., 2020; Moss et al., 2019).

### Population characteristics

For the purposes of this review, categorisations such as ‘ethnicity’, ‘immigrant’, and ‘racialised groups’, were extracted directly from the language used in each study. These definitions varied across jurisdictions and studies. For example, in Australia and New Zealand, studies focussed mainly on Indigenous populations. In Australia, Aboriginal or Torres Strait Islander status were a focus (Brophy et al., 2006; Kisely et al., 2004, 2015, 2020, 2021, 2023; Kisely, Preston, Xiao, Lawrence, Louise, & Crowe, 2013; Kisely, Preston, Xiao, Lawrence, Louise, Crowe, & Segal, 2013; Kisely & Xiao, 2018; Kisely, Xiao, Crowe, et al., 2014; Kisely, Xiao, Lawrence, & Jian, 2014; Ogilvie & Kisely, 2022; Segal et al., 2009, 2017; Suetani et al., 2022), whereas Māori communities were a focus in New Zealand (Beaglehole et al., 2021, 2023; Dey et al., 2021; Gibbs et al., 2004; Newton-Howes et al., 2014). Immigrant populations were a focus in 22 studies (Bardell-Williams et al., 2019; Brophy et al., 2006; Burgess et al., 2006; Kisely et al., 2004, 2015, 2020, 2021, 2023; Kisely, Preston, Xiao, Lawrence, Louise, & Crowe, 2013; Kisely, Preston, Xiao, Lawrence, Louise, Crowe, & Segal, 2013; Kisely & Xiao, 2018; Kisely, Xiao, Crowe, et al., 2014; Kisely, Xiao, Lawrence, & Jian, 2014; Lambert et al., 2009; Morandi et al., 2017; Moss et al., 2019; O’Brien et al., 2009; Ogilvie & Kisely, 2022; Parker et al., 2021; Segal & Burgess, 2006; Silva et al., 2019; Suetani et al., 2022), while 21 studies focussed on racialised groups (Barkhuizen et al., 2020; Beaglehole et al., 2021, 2023; Dey et al., 2021; Evans et al., 2010; Galon et al., 2012; Gibbs et al., 2004; Gonzales et al., 2015; Kisely, 2016; Mfoafo-M’Carthy, 2014; Monnery & Belgamwar, 2011; Newton-Howes et al., 2014; Paduret & Kelbrick, 2023; Patel et al., 2011; Segal et al., 2009; Segal et al., 2017; Swanson et al., 2009; Swartz et al., 2001, 2002; Van Dorn et al., 2006; Weich et al., 2020).

The sample sizes of the studies varied widely from 18 (Paduret & Kelbrick, 2023) to 24, 973 (Segal & Burgess, 2006) and related to the study methodology, with population-based studies having the largest.

### Relationship between immigrant, ethnicity and racialised groups and CTO placement and other forms of mandated community treatment

The objectives for each of the studies included in this review varied, and therefore there were mixed results between the relationship between ethnicity and CTOs.

Of the nine studies which had a primary focus on immigrant, ethnicity or racialised groups and CTO placement, six of these were quantitative studies (Galon et al., 2012; Kisely et al., 2020, 2021; Kisely & Xiao, 2018; Moss et al., 2019; Swanson et al., 2009). Two Australian studies had shown that being born in another country, increased the likelihood of CTO placement compared to being born in Australia (Kisely et al., 2013; Moss et al., 2019). Two other Australian studies showed that individuals from culturally or linguistically diverse backgrounds were more likely to be subjected to compulsory community treatment (Kisely et al., 2021, 2023). One American study had shown that people identifying as Black were five times more likely to be subject to outpatient commitment, but when adjusting for poverty, prevalence of mental illness, hospitalisation and outpatient mental health services use, this association was no longer present (Swanson et al., 2009). Another American study showed similar findings, reporting no significant differences in the rate at which OPC was applied to African Americans, the racialised group in this study (Galon et al., 2012).

Of the studies which did not directly focus on potential associations between immigrant, ethnicity or racialised groups and CTO placement, but presented data on this topic, findings were mixed (Bardell-Williams et al., 2019; Barkhuizen et al., 2020; Beaglehole et al., 2021, 2023; Brophy et al., 2006; Burgess et al., 2006; Dey et al., 2021; Evans et al., 2010; Gonzales et al., 2015; Kisely, 2016; Kisely et al., 2004, 2015, 2023; Kisely, Preston, Xiao, Lawrence, Louise, & Crowe, 2013; Kisely, Preston, Xiao, Lawrence, Louise, Crowe, & Segal, 2013; Kisely, Xiao, Crowe, et al., 2014; Kisely, Xiao, Lawrence, & Jian, 2014; Lambert et al., 2009; Monnery & Belgamwar, 2011; Morandi et al., 2017; O’Brien et al., 2009; Ogilvie & Kisely, 2022; Paduret & Kelbrick, 2023; Parker et al., 2021; Patel et al., 2011; Segal et al., 2009, 2017; Silva et al., 2019; Segal & Burgess, 2006; Suetani et al., 2022; Swartz et al., 2001, 2002; Van Dorn et al., 2006; Weich et al., 2020). One study found that there was a greater proportion of CTO cases among individuals born outside of Australia compared to controls (Kisely, Preston, Xiao, Lawrence, Louise, & Crowe, 2013). Another study reported that there was a greater proportion of individuals on CTOs who were born outside of Australia compared to controls, with similar proportions of Aboriginal or Torres Strait Islander identification between groups (Kisely et al., 2020). Subsequent research consistently found higher CTO rates among migrants and those from culturally and linguistically diverse backgrounds (Kisely et al., 2021, 2023). Similar patterns were reported in other studies (Bardell-Williams et al., 2019; Brophy et al., 2006; Burgess et al., 2006; Evans et al., 2010; Monnery & Belgamwar, 2011; Segal et al., 2017; Van Dorn et al., 2006). Other studies found that there were no differences in CTO placement amongst ethnic minority individuals (Beaglehole et al., 2023; Lambert et al., 2009; O’Brien et al., 2009; Parker et al., 2021; Suetani et al., 2022; Weich et al., 2020). While other studies found that being from an ethnic minority or racialised community did not increase rates of CTO placement, after adjusting for other variables (Barkhuizen et al., 2020; Silva et al., 2019; Swartz et al., 2002). Two studies had found that CTOs were more likely to be used in the care of service users of non-ethnic minority backgrounds (Kisely et al., 2020; Segal & Burgess, 2006). A cluster analysis revealed distinct profiles among CTO recipients: individuals in the ‘Connected’ cluster were more likely to be born outside Australia, and had shorter CTO durations, while the ‘Young males’ cluster included predominantly Australian-born males, with paranoid schizophrenia diagnoses (Brophy et al., 2006). Some studies, limited to descriptive data, also highlight cultural diversity. For example, in Canada, 17% of CTO recipients at two mental health centres reported neither English nor French as their first language, suggesting a significant proportion with diverse or immigrant backgrounds (O’Brien et al., 2009).

### Clinical outcomes for immigrants, ethnic minorities and racialised groups

Several studies have examined outcomes of people from ethnic, racialised and immigrant communities who were subject to CTOs.

One study reported variation in admission rates following CTO placement across ethnic groups: European/other and Māori heritage was associated with reduced admission frequency, while Pasifika background showed no significant change, and Asian ethnicity was associated with increased admissions (Beaglehole et al., 2021). Another study found that Aboriginal identity was associated with a greater risk of admission, and being born outside Australia also increased this risk (Kisely et al., 2004). In contrast, Aboriginal ancestry did not significantly affect hospital stay duration in another study (Segal et al., 2009).

Parker et al. (2021) found that 88.4% of participants who were subject to a CTO in the sample were born in Australia. In the group who remained on a CTO at discharge, 88.3% were born in Australia, and those who became voluntary at discharge, 88.9% were born in Australia. This shows that for those who were born in Australia, there was not much difference in those who were voluntary compared to those being subject to a CTO at discharge (Parker et al., 2021).

Beaglehole et al. (2023) had also reported on mortality data in a later study for patients placed on CTOs and found that people who were placed on CTOs who died from suicide were more likely to be of New Zealand European ethnic origin, and less likely to be Māori.

Access to specialised medical procedures showed no differences for those on CTOs who were born outside Australia or identified as Aboriginal or Torres Strait Islander (Kisely, Xiao, Lawrence, & Jian, 2014). Additionally, no significant differences in reported health and social function were observed between CTO cases and controls for these groups (Kisely, Xiao, Crowe, et al., 2014).

### Views and perceptions of service users towards CTOs

Three qualitative studies presented data on views and perceptions towards CTOs (Gibbs et al., 2004; Mfoafo-M’Carthy, 2014; Newton-Howes et al., 2014). Newton- Howes et al. (2014) explored the views of Māori and non-Māori individuals in Hawke’s Bay, New Zealand and found there were no differences in the views of each group regarding CTOs (Newton-Howes et al., 2014). Gibbs et al. (2004) also explored the impacts of CTOs on Māori individuals but also taking into consideration their whanau (extended family), and the views they had of mental health professionals. Both benefits and drawbacks of CTOs were identified for Māori service users and their whanau. While CTOs were seen as improving safety, access to services and offering an alternative to hospital or homelessness, drawbacks included the sense of external control, particularly over medication and personal choices (Gibbs et al., 2004). Mfoafo-McCarthy (2014) conducted semi-structured interviews with ethnic minority individuals who had been subject to CTOs in Toronto, Canada. Participants perceived both negative and positive impacts of CTOs, but they generally viewed CTOs as the best option available to them at the time. However, a smaller group believed that CTOs were racially biased and felt that their racialised identities played a role in the treatment they experienced (Mfoafo-M’Carthy, 2014).

Two quantitative studies examined the perceptions of service users (Swartz et al., 2002; Van Dorn et al., 2006). White people were positively associated with mandated-related barriers to care amongst patients subjected to mandated community treatment. These ‘mandated-related barriers to care’ were identified when patients responded ‘yes’ or ‘no’ to questions such as whether they refused help for mental health, alcohol or drug problems due to concerns about being forced into taking unwanted medications or treatment or fear of being under involuntary commitment (Van Dorn et al., 2006). However, another study reported higher levels of perceived coercion among African Americans (Swartz et al., 2002).

## Discussion

This scoping review is the first to comprehensively review and synthesise the literature on CTOs and other forms of compulsory community treatment, with a specific focus on associations with immigrant, racialised and ethno-culturally diverse communities. Between 2001 and 2023, only nine studies directly investigated the association between these groups and CTOs (Galon et al., 2012; Gibbs et al., 2004; Kisely et al., 2020, 2021; Kisely & Xiao, 2018; Mfoafo-M’Carthy, 2014; Moss et al., 2019; Newton-Howes et al., 2014; Swanson et al., 2009). Among these nine studies, findings were mixed, varying by study design, population and jurisdiction. Additionally, 34 other studies presented data on this topic but did not directly focus on these associations (Bardell-Williams et al., 2019; Barkhuizen et al., 2020; Beaglehole et al., 2021, 2023; Brophy et al., 2006; Burgess et al., 2006; Dey et al., 2021; Evans et al., 2010; Gonzales et al., 2015; Kisely, 2016; Kisely et al., 2004, 2015, 2023; Kisely, Preston, Xiao, Lawrence, Louise, & Crowe, 2013; Kisely, Preston, Xiao, Lawrence, Louise, Crowe, & Segal, 2013; Kisely, Xiao, Crowe, et al., 2014; Kisely, Xiao, Lawrence, & Jian, 2014; Lambert et al., 2009; Monnery & Belgamwar, 2011; Morandi et al., 2017; O’Brien et al., 2009; Ogilvie & Kisely, 2022; Paduret & Kelbrick, 2023; Parker et al., 2021; Patel et al., 2011; Segal et al., 2009, 2017; Silva et al., 2019; Segal & Burgess, 2006; Suetani et al., 2022; Swartz et al., 2001, 2002; Van Dorn et al., 2006; Weich et al., 2020). Many of these studies appropriately adjusted for ethnicity and immigration history as covariates rather than focussing on them as the primary exposure of interest.

The current body of literature on this topic indicates that the relationship between ethnicity and CTOs is complex. Some studies reported an increased likelihood of CTO placement among individuals from ethnic minority backgrounds (Burgess et al., 2006; Evans et al., 2010; Kisely et al., 2015, 2020, 2021, 2023; Kisely, Preston, Xiao, Lawrence, Louise, & Crowe, 2013; Kisely, Preston, Xiao, Lawrence, Louise, Crowe, & Segal, 2013; Kisely & Xiao, 2018; Monnery & Belgamwar, 2011; Moss et al., 2019; Ogilvie & Kisely, 2022; Segal & Burgess, 2006; Swartz et al., 2001; Weich et al., 2020), while others found no such association (Bardell-Williams et al., 2019; Barkhuizen et al., 2020; Dey et al., 2021; Galon et al., 2012; Gonzales et al., 2015; Lambert et al., 2009; Morandi et al., 2017; Paduret & Kelbrick, 2023; Parker et al., 2021; Patel et al., 2011; Segal et al., 2017; Silva et al., 2019; Swanson et al., 2009; Suetani et al., 2022).

These mixed findings are likely due in part to the substantial heterogeneity across studies, including differences in study design, methods, jurisdictional variations in compulsory community treatment, as well as baseline population demographics and the differential risk of developing a mental disorder based on ethnic group membership and immigration history.

It is important to note that most of the literature on this topic comes from Australia and New Zealand, with indigenous populations, (such as Aboriginal, Torres Strait and Māori ethnicity), being studied in Australia (Bardell-Williams et al., 2019; Beaglehole et al., 2021, 2023; Brophy et al., 2006; Burgess et al., 2006; Dey et al., 2021; Gibbs et al., 2004; Kisely et al., 2004, 2015, 2020, 2021, 2023; Kisely, Preston, Xiao, Lawrence, Louise, & Crowe, 2013; Kisely, Preston, Xiao, Lawrence, Louise, Crowe, & Segal, 2013; Kisely & Xiao, 2018; Kisely, Xiao, Crowe, et al., 2014; Kisely, Xiao, Lawrence, & Jian, 2014; Lambert et al., 2009; Morandi et al., 2017; Moss et al., 2019; Newton-Howes et al., 2014; Ogilvie & Kisely, 2022; Parker et al., 2021; Segal & Burgess, 2006; Segal et al., 2009, 2017; Suetani et al., 2022). Some studies had found that indigenous populations were more likely to be subject to compulsory community treatment (Kisely et al., 2023), and CTO-related outcomes while being placed on a CTO (Kisely et al., 2021). However, other studies had found that being of an indigenous status was not a significant factor for CTO use, when controlling for other variables (Kisely et al., 2015, 2021, 2023; Ogilvie & Kisely, 2022; Segal et al., 2017; Suetani et al., 2022) or CTO-related outcomes (Dey et al., 2021; Kisely, Xiao, Crowe, et al., 2014; Kisely, Xiao, Lawrence, & Jian, 2014; Segal et al., 2009). One study from New Zealand had even shown Māori ethnicity had a reduced admission frequency while on CTOs (Beaglehole et al., 2021). Considering the history of coercion and systematic discrimination Indigenous communities have faced (Gibbs et al., 2004; Kisely et al., 2017, 2023), focus on Indigenous and Māori people in relation to is important to consider CTOs. This allowed for a better understanding of how CTOs may impact these populations, shedding light on potential disparities in treatment and the need for culturally sensitive approaches to mental health care and equitable service delivery. Future research can use these studies as a model to focus on Indigenous populations in other settings, as well as marginalised immigrant, ethnic and racialised communities.

Beyond quantitative studies, a small but important body of qualitative literature offers valuable insights (Gibbs et al., 2004; Mfoafo-M’Carthy, 2014; Newton-Howes et al., 2014). Given the diversity of experiences, it is unsurprising that studies exploring the perspective of minoritised and racialised individuals on CTOs revealed mixed findings. For example, Newton-Howes et al. reported no differences between Māori and non-Māori perspectives on CTOs (Newton-Howes et al., 2014). In contrast, studies by Gibbs et al. and Mfoafo-M’Carthy highlighted both positive and negative experiences, including benefits such as increased patient safety and access to services, alongside concerns about ethnoracial-related inequities, inadequate treatment and a sense of imposed external control, particularly around medication and decision-making (Gibbs et al., 2004; Mfoafo-M’Carthy, 2014). These findings suggest that ethnicity may play a role in shaping how individuals experience CTO placement, though the impact may vary between individuals. These insights are important, and further qualitative research can help inform avenues for future research.

### Strengths of this review

One of the strengths of this scoping review is the breadth and diversity of literature included, as it maps the current literature across different contexts and jurisdictions. The inclusion of both qualitative and quantitative research provides deeper insights into the lived experiences of people and communities who have experiences with CTOs, while also examining population-based health administrative data findings. Additionally, the use of comprehensive terminology for compulsory treatments ensured that a wide range of relevant studies, across different jurisdiction, were included, capturing studies where different terms for compulsory care are used. Furthermore, the descriptive rather than interpretive nature of our extraction fields reduced subjectivity, ensuring a more objective approach to data extraction.

### Limitations to the current review

The restriction to English language studies may excluding relevant findings published in other languages (e.g. potential studies from non-English jurisdictions such as Switzerland, Italy, France, Spain and Quebec, Canada; Neimann Rasmussen & Montgomery, 2018). The inclusion of studies from a variety of jurisdictions and forms of compulsory treatment may have introduced heterogeneity, complicating the interpretations of findings across different contexts. Local, jurisdiction-specific studies may be important in understanding these relationships of interest moving forward.

Considering only nine studies focussed directly on the association between CTOs and specific populations, interpretation of data from the additional studies, particularly in relation to potential associations between specific groups and CTOs must be done with caution. There is a risk of a ‘Table 2 fallacy’ occurring, whereby adjusted associations may be misinterpreted as primary exposure effects. For instance, some studies adjusted for ethnicity, immigration status or racialised groups but did not focus on these as primary exposures (Westreich & Greenland, 2013). To reduce this risk, we have divided the studies accordingly based on this to aid interpretation. However, any observed associations between ethnicity and CTO outcomes in the studies which did not directly focus on this relationship should be interpreted with caution. This underscores the importance of future studies explicitly designed to examine ethnicity, immigration status or racialised identity as primary exposures of interest, using robust methods that can account for confounding variables without obscuring their role. While the availability of data to adjust for these factors is promising, the need for research that places equity questions at the core, rather than addressing them as secondary considerations.

Additionally, this review relied on a single screener for study selection, which may have introduced selection bias and the risk of missed studies. Although scoping review frameworks do not require dual screening, the absence of a second reviewer increases the possibility of relevant studies being inadvertently excluded. To mitigate this, screening decisions were guided by predefined eligibility criteria, and discussions were held where study inclusion was unclear. However, future reviews may benefit from dual screening to enhance reliability and ensure all relevant studies are captured. Similarly, data extraction was conducted primarily by a single reviewer, which may introduce subjectivity and potential inconsistencies in the interpretation of study findings. While a second reviewer was involved in full-text screening, and discrepancies were resolved through discussion and, when necessary, consultation with a third reviewer, the absence of dual independent data extraction may have increased the risk of data omission or misinterpretation. Future studies could strengthen methodological rigour by implementing independent data extraction by multiple reviewers to improve accuracy and reduce potential bias in the synthesis of findings.

### Strengths of the published literature

Many studies appropriately adjusted for ethnicity, immigrant status, recognising these as this as potential confounding variables that could bias findings (Bardell-Williams et al., 2019; Barkhuizen et al., 2020; Beaglehole et al., 2021, 2023; Brophy et al., 2006; Burgess et al., 2006; Dey et al., 2021; Gibbs et al., 2004; Kisely, 2016; Kisely et al., 2004, 2015, 2020, 2021, 2023; Kisely, Preston, Xiao, Lawrence, Louise, & Crowe, 2013; Kisely, Preston, Xiao, Lawrence, Louise, Crowe, & Segal, 2013; Kisely & Xiao, 2018; Kisely, Xiao, Crowe, et al., 2014; Kisely, Xiao, Lawrence, & Jian, 2014; Lambert et al., 2009; Mfoafo-M’Carthy, 2014; Morandi et al., 2017; Moss et al., 2019; Newton-Howes et al., 2014; O’Brien et al., 2009; Ogilvie & Kisely, 2022; Paduret & Kelbrick, 2023; Parker et al., 2021; Patel et al., 2011; Segal et al., 2009, 2017; Silva et al., 2019; Suetani et al., 2022; Weich et al., 2020). Additionally, studies have examined whether ethnic minority status while being subject to a CTO is associated with outcomes, such as access to specialised medical procedures (Kisely, Xiao, Lawrence, & Jian, 2014), broader health and social functioning (Kisely, Xiao, Crowe, et al., 2014) and the effectiveness of CTOs in reducing inpatient admissions (Beaglehole et al., 2021). These studies illustrate the multifaceted ways in which ethnicity and CTOs can intersect and implications for broader outcomes. Further qualitative research with individuals and communities may also help clarify what outcomes are important to focus on in relation to CTOs.

### Limitations of prior research

Only three qualitative studies were included (Gibbs et al., 2004; Mfoafo-M’Carthy, 2014; Newton-Howes et al., 2014) highlighting a notable gap in research focussing of service users and communities. This represents a clear opportunity for further exploration in future studies.

Additionally, while the majority of studies in this review originate from high-income Western countries and offer valuable insights into specific jurisdictions, it also highlights a broader structural gap in the global literature. Research on CTOs and compulsory community treatment from low- and middle-income countries (LMICs) is virtually absent, despite the fact that coercive psychiatric practices, legal frameworks, and community-based mental health models may differ substantially across socio-political and economic contexts. This lack of representation not only limits the generalisability of findings but underscores the need for deliberate efforts to include diverse global perspectives. Research from LMICs could offer important insights into how systemic factors – such as access to care, stigma, legal protections and resource limitations – intersect with race, ethnicity and migration status in shaping experiences of compulsory community treatment. Future research should therefore prioritise contextually grounded studies from underrepresented regions to build a more globally inclusive understanding of CTOs and their implications.

There is limited data on specific ethnic groups in relation to CTOs, including South and East Asian populations and Hispanic/Latino populations. This gap may reflect the demographics of the jurisdictions studied. Many studies grouped multiple ethnicities into broad categories such as ‘other’ or ‘culturally or linguistically diverse background’, obscuring data for individual minority ethnic groups which may have different experiences and outcomes. Therefore, addressing these distinctions is crucial to understanding the unique impacts of CTOs across different communities.

Although most of the studies explicitly presented data on ethnicity, immigrant status or racialised groups there were a few cases where this was not explicitly but rather categorised participants as not speaking the official language (suggesting a diverse, immigrant population; O’Brien et al., 2009). Such data, and further examination of who these participants may be, may also provide valuable insights into the relationship between minority-service users and CTO use.

Of note, there is a risk of a Table 2 fallacy, whereby adjusted associations may be mistakenly interpreted in the same manner as the primary exposure when models are adjusted for ethnicity, racialised groups or immigration status. This analytical limitation reduces the capacity to draw strong conclusions about causal relationships or disparities. Future research should intentionally frame these variables as core exposures and build designs capable of exploring their interaction with structural, clinical and contextual factors.

### Future directions

Based on the findings of this review, there is ample opportunities for future research to focus directly on the relationship between ethnicity and CTOs to enhance understanding of what is a complex and understudied relationship. Greater focus on specific ethnic groups, such as South and East Asian or Hispanic/Latino populations, is particularly needed.

Future research may also benefit from examining contextual factors and may consider the impact of ethnic density (the proportion of people from a specific ethnic group within a defined area) and its relationship with CTO use, as prior studies suggest a possible link (Das-Munshi et al., 2012; Rotenberg et al., 2017). Additionally, quantitative studies beyond Australia, New Zealand and Canada – the regions where studies have occurred thus far – could offer important insights.

Future research should also consider how to directly account for structural factors that might impact care at baseline, as ethnic minority individuals are more likely to experience racism and discrimination in the mental health system, which may influence CTO use. Considering the impacts of area-level marginalisation on service use and risk of mental illness (Rotenberg et al., 2022), as well as ethnic density effects (Das-Munshi et al., 2012), might be avenues where lines of inquiry relating to structural factors might begin.

## Conclusions

This scoping review mapped the findings from the existing literature regarding the relationship between ethnicity and community treatment orders (CTOs). The relationship between ethnicity and CTOs are mixed and there is substantial heterogeneity between studies. There are ample opportunities for future research to directly focus on the relationship between ethnicity and CTOs in a wider range of countries and among diverse minority ethnic groups to inform equitable mental health care practices.

In light of the evidence and gaps identified, it is essential for future clinical and policy approaches to prioritise culturally safe, non-coercive and community-led alternatives to CTOs. This may include involving family members or trusted community leaders in care planning, integrating peer workers with lived experience into mental health teams and ensuring access to high-quality interpretation services.

Health systems should also implement culturally safe assessment tools, provide anti-racism and structural competency training for clinicians and collaborate with racialised and minoritised communities to co-develop care models that are respectful, inclusive and responsive to community needs. These strategies have the potential to reduce reliance on coercive practices and promote more just and person-centred mental health care.

## Figures and Tables

**Figure A2.**
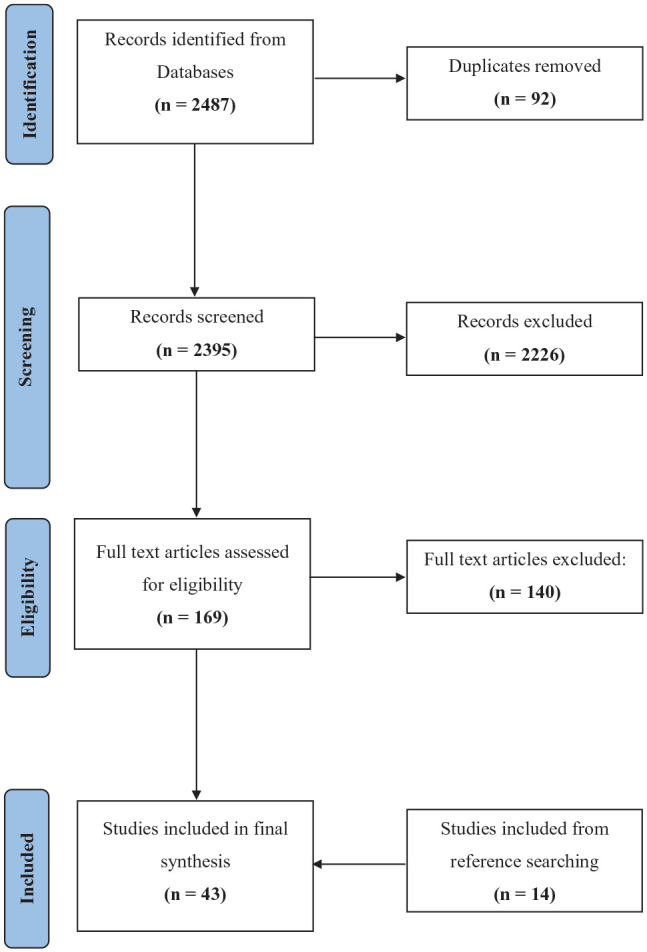
Search outcomes and scoping review flow.

## Appendix B

**Table B1.** Data extraction table of included studies. *Studies with a primary focus on Immigrant, Ethnicity or Racialised groups*.

Author, year, country	Form of compulsory/mandated community treatment	Study objectives	Study setting, data sources and/or measures	Study design	Population/sample size	Immigrant, Ethnicity or Racialised group	Relevant findings
Galon et al. (2012, (USA)	Outpatient Commitment (OPC)	To examine the frequency of use of OPC and Assertive Community Treatment (ACT) and compares their influence on the perceptions of procedural justice/choice and coercion/negative pressure on African Americans and White people.	Study measures included: Admission Experience Survey (AES), the Ohio Consumers Mental Health Outcomes Survey, and the Community Violence and Victimisation Scale.	Secondary analysis of a quasi-experimental study	*n* = 154	African American, White	There were no significant differences in the rate at which OPC or ACT was applied to African Americans. Procedural justice/choice contributed significantly to the perception of coercion/negative pressure in the OPC and ACT groups but had no effect on African Americans.
Gibbs et al. (2004, New Zealand)	Community Treatment Order (CTO)	To consider the impact of community treatment orders (CTOs) on Māori patients and their whanau (extended family) and associated views of mental health professionals.	Detailed semi structured interviews with people subject to community treatment orders in Otago, New Zealand and with those most concerned with their care.	Qualitative study	*n* = 39	Māori	Both benefits and drawbacks of CTOs were identified for Māori service users and their whanau. CTOs were considered helpful in increasing safety and whanau security as well as promoting access to services. They were favoured over hospital care, forensic care and homelessness. The drawbacks included the sense of external control imposed on both Māori people and staff, particularly concerning medication and restrictions on choices.
Kisely and Xiao (2018, Australia)	Community Treatment Order (CTO)	To investigate if the likelihood of being subject to a CTO differs between Australian- and foreign-born people in Western Australia.	Linked health administrative data from Western Australia	Retrospective cohort study	2,958 CTO cases and 2,958 controls (*n* = 5,916)	Country of birth, Region of birth	People born in any country in the world other than New Zealand, the UK or Ireland were more likely to be placed on a CTO compared to people born in Australia.
Kisely et al. (2020, Australia)	Compulsory Community Treatment (including both Community Treatment Orders (CTO) and Forensic Orders (FO)).	To investigate whether people from Indigenous or culturally and linguistically diverse backgrounds (based on country of birth, or preferred language) were more likely to be subject to compulsory community treatment and to assess the impact of compulsory community treatment on health service use over the following 12 months.	Linked health administrative data from Western Australia	Retrospective cohort study	7,432 cases and 7,432 controls (total *n* = 14,864)	Indigenous Australia, Country of birth outside of Australia, NZ, UK, Ireland and North America; Preferred language other than English	Indigenous Queenslanders and people coming from a culturally and linguistically diverse background were more likely to be subject to compulsory community treatment.
Kisely et al. (2021, Australia and New Zealand)	Community Treatment Order (CTO)	To assess the health service, clinical and psychosocial outcomes of compulsory community treatment in Australia and New Zealand and to explore if culturally and linguistically diverse, Indigenous status or other factors predict community treatment orders.	PubMed/Medline, Embase, CINAHL and PsycINFO were searched from inception to January 2020.	Systematic Review and Meta-Analysis	31 publications from 12 studies, 24 publications included in meta-analysis, approximately 53,170 cases and 164,864 controls (*n* = 218, 034)	Culturally and Linguistically Diverse Background (CALD)/migrant background, Indigenous status	Those from a culturally and linguistically diverse or migrant background were nearly 40% more likely to be subject to a community treatment order. Indigenous status was not associated with community treatment order placement in Australia and there were no data New Zealand for Indigenous peoples.
Mfoafo-M’Carthy (2014, Canada)	Community Treatment Order (CTO)	To present findings from research on the lived experiences of people from ethnic minority backgrounds who have been subjects to CTOs in Toronto, Canada, and their perceptions of its impact on their lives.	Semi-structured interviews	Qualitative study	*n* = 24	Black Canadians, African Canadians, Caribbean Canadians, East Asian, Middle East	Participants perceived both positive and negative impacts of CTOs. They generally viewed CTOs as the best option available to them at that time. However, a smaller group believed that CTOs were racially biased and felt that their race played a role in the insufficient treatment and support they experienced.
Moss et al. (2019, Australia)	Community Treatment Order (CTO) and Forensic Order (FO)	To compare the use of community treatment orders (CTOs) and forensic orders (FOs) in a culturally and linguistically diverse (CALD) population to that in a non-CALD population.	Linked health administrative data from the Metro South Health Area, Brisbane, Queensland, Australia.	Retrospective cohort study	*n* = 976	Country of birth: Australia, New Zealand, England, Vietnam. . ., Languages spoken requiring an interpreter: Vietnamese, Mandarin, Arabic and Farsi. . . .	Use of compulsory community treatment (CTOs and FOs) was similar for those born in Australasia, British Isles, North America and Europe but significantly higher for those born elsewhere. The use of an interpreter significantly increased the likelihood of compulsory community treatment.
Newton-Howes et al. (2014, New Zealand)	Community Treatment Order (CTO)	To compare the views of Māori and non-Māori with respect to CTOs.	Focus groups, the local Kaupapa Māori mental health service: Te Taiwhenua O Heretaunga (TTWOH) Hawke’s Bay, New Zealand	Qualitative study	*n* = 79	Māori, non-Māori	There were no differences in the views of Māori compared to non-Māori people with respect to compulsory community treatment. There were no differences in the views of Māori cared for by mainstream compared to culturally specialist Māori mental health service.
Swanson et al., (2009, USA)	Involuntary Outpatient Commitment	To explore racial disparities in involuntary outpatient commitment	Heath administrative data from the New York State Office of Mental Health (OMH) administrative and clinical records on people receiving court-ordered treatment under Kendra’s Law from 6 counties	Cross-sectional study	*n* = 893	Black, White	People identified as Black were approximately five times more likely to be subject to outpatient commitment However, this association is no longer present after adjusting for poverty, prevalence of mental illness, and hospitalisation as well as outpatient mental health services use. Broader social and systemic variables are likely what are driving disparities.

**Table:** *Studies that presented data on Immigrant, Ethnicity or Racialised groups (but not as a primary study focus)*.

Author, year, country	Form of compulsory/ mandated community treatment	Study objectives	Study setting, data sources and/or measures	Study design	Population/sample size	Immigrant, ethnicity or racialised group	Relevant findings
Bardell-Williams (2019, Australia)	Community Treatment Order (CTO)	To determine the rates, determinants and outcomes associated with the use of CTOs in young people with a first episode of psychosis (FEP).	Medical records from the Orygen Youth Health (OYH) – a mental health service for young people aged between 15 and 24 years, as well as clinical measure assessing symptoms and functioning	Retrospective cohort study	*n* = 544	Place of birth: Born in Australia, First generation migrant	67.4% of participants were born in Australia and 25.1% were first-generation migrants, however there was not a statistically significant association between place of birth and being subject to a CTO.
Barkhuizen et al. (2020, UK)	Community Treatment Order (CTO)	To compare clinical outcomes between patients placed on CTOs to a control group of patients discharged to voluntary community mental healthcare.	Deidentified electronic health records of mental health services provided by the South London and Maudsley National Health Service (NHS) Foundation Trust (SLaM).	Retrospective cohort study	830 subjects and 3659 controls, (*n* = 4489).	Black, White, Other	In an unadjusted analysis, Black patients had a greater odd of being subject a CTO compared to white patients. However, this association was not significant after adjusting for other factors like forensic, clinical, and demographic characteristics, indicating that these factors might explain the initial observed association between ethnicity and CTO placement.
Beaglehole et al. (2021, New Zealand)	Compulsory Community Treatment Order (CTO)	To examine the effectiveness of CTOs using routine data in the New Zealand context.	Programme for the Integration of Mental Health Data (PRIMHD) dataset records	Retrospective cohort study	*n* = 14,726	European/Other, Māori, Pasifika, Asian	European/other and Māori ethnicity were associated with a reduced admission frequency on CTOs, whereas there was no significant change in admission frequency for people of Pasifika ethnicity. Asian ethnicity was associated with increased admissions on CTOs.
Beaglehole et al. (2023, New Zealand)	Compulsory Community Treatment Order (CTO)	To report mortality for patients placed on CTOs and analyse data according to CTO status, mortality cause and diagnosis.	Individuals were identified in the Programme for the Integration of Mental Health (PRIMHD) dataset. The underlying cause of death was provided by the Registrar of Births, Deaths and Marriages to the Ministry of Health.	Retrospective cohort study	*n* = 14 726 patients (and 1328 deaths)	New Zealand European, Māori	It was found that patients who were placed on CTOs who died from suicide were more likely to be of New Zealand European ethnicity (67.4% New Zealand European ethnicity) than the other groups, and less likely to be Māori (20.2% Māori ethnicity).
Brophy et al. (2006, Australia)	Community Treatment Order (CTO)	To explore the clinical, social and demographic characteristics of 164 people on Community Treatment Orders (CTOs) in one area mental health service in Victoria, Australia. The results of this study are presented to address the question of whether people on Community Treatment Orders can be categorised into statistically reliable, qualitatively distinct groupings.	The Mental Health Client Management Interface (CMI)	Descriptive Cross-sectional study and Exploratory Cluster Analysis	*n* = 164	Country of birth: Australia, overseas	Considerable diversity in country of birth was represented in this client group (31% were born outside Australia) and this reflects the diversity of the catchment area. Only one person was recorded as being Aboriginal or Torres Strait islander. People in Cluster 1 (*Connected*) were more likely to be born outside of Australia, and people in Cluster 2: (*Young males*) were most likely to be born in Australia
Burgess et al. (2006, Australia)	Community Treatment Order (CTO)	To test the hypothesis that hospital discharges of people who are subject to CTOs are associated with a reduced risk of readmission.	The Victorian Psychiatric Case Register (VPCR)	Retrospective cohort study	16,216 discharges subject to a CTO and 112,211 not subject to a CTO (*n* = 128,427)	Country of birth: Australia, English-speaking country, non-English speaking country, Unknown	Patients on CTOs were typically younger, single, and more likely born outside Australia. While CTOs did not reduce the risk of readmission, the characteristics of patients under CTOs changed over time.
Dey et al. (2021, New Zealand)	Community Treatment Order (CTO)	To document the sociodemographic and clinical variables associated with discharge under compulsory treatment orders in patients with schizophrenia or related disorders. from a teaching hospital which serves roughly 10% of New Zealand’s population.	Clinical files of patients discharged from an adult (age 18–65) inpatient psychiatric unit with diagnoses of schizophrenia or related disorders.	Retrospective cohort study	*n* = 200 (from 349 discharged patients)	Māori, non-Māori	Although Māori ethnicity people were overrepresented in the sample, ethnicity was not associated with CTO placement. Māori ethnicity was not a significantly contributing factor to rehospitalisation for CTOs.
Evans et al. (2010, UK)	Supervised Community Treatment	To describe the first 6 months of newly introduced community treatment orders (CTOs) at a mental health service in Birmingham and describe patterns of use and early outcomes.	Electronic health records from Birmingham and Solihull Mental Health NHS Foundation Trust.	Retrospective Clinical Audit	*n* = 104	White, Asian, Black, Other	When compared to the demographics of the local area, Black and minority ethnic groups were overrepresented in being subject to Supervised Community Treatment.
Gonzales et al. (2015, USA)	Assisted Outpatient Treatment	To compare Assisted Outpatient Treatment (AOT) eligibility characteristics among participants receiving community treatment through AOT and non-AOT referrals.	Charts were reviewed from three Assertive Community Treatment teams within one treatment agency in New York City. Intake information was coded for AOT eligibility information, suicide history, and risk of future violence according to the Historical Clinical Risk Management−20, version 3.	Cross-sectional study	*n* = 131	African American, White/European American, East Asian, Hispanic/Latino, Arab/Middle Eastern, Other	No significant differences in AOT were found with respect to ethnocracies background.
Kisely (2016, Canada)	Community Treatment Order (CTO)	To summarise the evidence from studies conducted in Canada regarding whether CTOs can reduce health service use or improve clinical and social outcomes for people with severe mental illness.	A comprehensive search of PubMed and MEDLINE (March 2015)	Systematic review	19 publications, 9 publications from 8 studies included in the review, *n* = 746+	Ethnic minority individuals: Black Canadians, African Canadians or Caribbean Canadians, the rest being from Asia or the Middle East	Out of the 9 papers from 8 studies included in this systematic review, one focussed on ethnicity and CTOs. The result of this paper is listed elsewhere.
Kisely et al. (2004, Australia)	Community Treatment Order (CTO)	To examine whether CTOs reduce admission rates, taking socio-demographic factors, clinical factors, case complexity and previous psychiatric and forensic history into account	Linked health administrative data from Western Australia	Retrospective cohort study	265 CTO cases with 265 matched controls and 224 consecutive controls (*n* = 754)	Aboriginality, Country of birth (Not Australia)	Apart from being on a CTO, aboriginal ethnicity was associated with a significantly greater risk of admission. Being born outside of Australia was also associated with an increased risk of admission, but only slightly.
Kisely et al. (2013, Australia)	Community Treatment Orders (CTO)	To examine whether outcomes change as clinicians gain experience in the use of community treatment orders (CTOs).	Linked health administrative data from Western Australia	Retrospective cohort study	2958 CTO cases and controls (*n* = 5,916)	Country of birth: Australia, Aboriginal or Torres Strait status	CTO cases were less likely to be born in Australia. Those who were Indigenous had reduced outpatient contact following CTO placement.
Kisely et al. (2013, Australia)	Community Treatment Order (CTO)	To investigate whether CTOs could reduce all-cause mortality among patients with psychiatric disorders.	Linked health administrative data from Western Australia	Retrospective cohort study	2958 CTO cases and controls (*n* = 5,916)	Country of birth outside of Australia, Aboriginal or Torres Strait status	Those in the CTO group were more likely to be born outside of Australia compared to the controls. The likelihood of identifying as Aboriginal or Torres Strait Islander was the same for both the CTO group and the control group.
Kisely et al. (2014 Australia)	Community Treatment Order (CTO)	To examine whether being subject to a CTO, had better outcomes on the Health of the Nation Outcome Scales (HoNOS; a 12-item scale which is used to assess the health and social functioning of people with severe mental illness).	Linked health administrative data from Western Australia	Retrospective cohort study	1296 CTO cases and 1296 controls (*n* = 2,592)	Country of birth: Australia, Aboriginal or Torres Strait status	No significant difference in HoNOS scores between CTO cases and controls at six- and twelve-month follow-up for those born outside of Australia or those of Aboriginal or Torres Strait status.
Kisely et al. (2014, Australia)	Community Treatment Order (CTO)	To investigate whether the reduction in preventable deaths from physical disorders due to initiation of compulsory community treatment was mediated by better access to specialised medical procedures.	Linked health administrative data from Western Australia	Retrospective cohort study	2757 people subject to compulsory treatment and 2687 control subjects (*n* = 5444).	Country of birth outside of Australia, Aboriginal or Torres Strait status	Compulsory community treatment did not result in greater access to specialised procedures at all time points, including for those born outside of Australia or those of Aboriginal or Torres Strait status.
Kisely et al. (2015, Australia)	Community Treatment Order (CTO)	To investigate factors associated with CTO placement in Western Australia and see if there were any changes over the 11 years following their introduction.	Linked health administrative data from Western Australia	Retrospective cohort study	2958 patients on CTOs and 2832 controls (*n* = 5,790).	Country of birth outside of Australia, Aboriginal or Torres Strait status	People born outside Australia were more likely to be subject to a CTO. There was no association between Indigenous background and being subject to a CTO.
Kisely et al. (2023, Australia and New Zealand)	Community Treatment Order (CTO)	To assess whether factors associated with CTO placement or subsequent outcomes vary by rates of use.	A systematic search of PubMed/Medline, Embase, CINAHL, the Cochrane Central Register of Controlled Trials and PsycINFO	Systematic review and Meta-Analysis	35 publications from 16 studies, 29 publications included in the meta-analysis, approximately 56 541 subjects and 170 156 controls (*n* = 226,697)	Indigenous or Culturally and Linguistically Diverse (CALD) background	Those from immigrant backgrounds were 47% more likely to be on an order. Those from a migrant or CALD background were found to be nearly 40% more likely to be on an order. In Australia, Indigenous status was not associated with CTO use (no data from New Zealand).
Lambert et al. (2009, Australia)	Community Treatment Order (CTO)	To investigate the relationship between CTOs and LAI use in patients with schizophrenia.	North- Western Mental Health Programme (NWMHP) central administrative database. central database.	Retrospective cohort study	*n* = 1,369	English-speaking country of birth, non-English-speaking background country of birth	A similar percentage of people from an English-speaking background were placed on a CTO (12.8%) compared to those from a non-English speaking background (11.6%).
Monnery and Belgamwar (2011, UK)	Supervised Community Treatment (SCT), Community Treatment Order (CTO)	To investigate whether new CTOs have been applied within the North Staffordshire Combined Healthcare NHS Trust. According to guidelines, and to examine the reasons behind use and their impact on admission to hospital.	The medical notes of all patients subject to SCT since its commencement in 2008 using a data collection proforma.	Retrospective cohort study	*n* = 42	African Caribbean and Black; Asian, including Indian, Pakistani, Bangladeshi, British Asian and other Asian; White British	There was an over-representation of Black and African Caribbean and Asian groups among patients in the study compared with the general population in the catchment area of the North Staffordshire Combined Healthcare Trust.
Morandi et al. (2017, Australia)	Community Treatment Order (CTO)	To determine CTO frequency in a large representative sample of first episode psychosis (FEP) patients, and to compare the characteristics of patients with or without CTO before entry, during treatment and at discharge from an early psychosis programme in Melbourne, Australia	Medical files (based on a previously conducted file audit study (‘FEPOS’)	Retrospective cohort study	*n* = 660	Born in Australia; Region of birth: Oceania, Europe, Africa and Middle East, Asia	No differences in CTO placement among FEP patients based on ethnicity.
O’Brien et al. (2009, Canada)	Community Treatment Order (CTO)	To examine the association of community treatment orders (CTO) with community engagement and housing arrangements for outpatients from a mental health hospital in Ontario, Canada.	All patients issued CTOs from the Royal Ottawa Mental Health Centre and the Montfort Hospital using a standardised information collection tool meeting ministry of health and long-term care reporting requirements.	Retrospective cohort study	*n* = 84	English or French as a first language.	67% reported English as their first language, 16% French and 17% reported neither English nor French as their first language. Neither English nor French background is suggestive of a diverse cultural background, potentially immigrant.
Ogilvie and Kisely (2022, Australia)	Community Treatment Order (CTO)	To investigate the characteristics and outcomes of people placed on CTOs.	Health administrative data: The Queensland Cross-sector Research Collaboration (QCRC) repository	Retrospective cohort study	211 CTO cases, 413 controls on voluntary treatment (*n* = 624)	Indigenous status	Indigenous Australians were significantly over-represented in the CTO group. When accounting for other factors altogether, Indigenous status was no longer significant.
Paduret and Kelbrick (2023, UK)	Community Treatment Order (CTO)	To identify the rate of CTO, use in a typical NHS Early Intervention in Psychosis (EIP) service, to identify the typical patient profile (demo- graphic and clinical characteristics) of those subject to CTOs; and to examine time spent subject to CTOs, and CTO outcomes, including relapse rates (defined as the number of hospital admissions in the 12-month period following CTO start).	The rs of patients subject to CTOs while in the Northamptonshire EIP service, Northamptonshire, England, UK and service over the period between 2019 and 2021.	Retrospective chart review	*n* = 18	White British, other White European, Black African, African-Caribbean, Asian, mixed.	The distribution of ethnic groups within this sample was representative of that of Northamptonshire.
Parker et al. (2021 Australia)	Community Treatment Order (CTO)	To compare the post-discharge outcomes of people admitted to community-based residential mental health rehabilitation facilities in Queensland and subject to a CTO and who either had the order discontinued or not prior to discharge	Administrative data for admissions to five Community Care Units (CCU) sites from electronic health databases by the Queensland Health Data Linkage Unit	Retrospective cohort study	63 people whose status changed to voluntary (CTO discontinued), 248 people who remained on CTOs (*n* = 311)	Country of birth (Australia)	It was found that 88.4% of participants in the sample who were subject to a CTO were born in Australia. In those subject to a CTO at admission and were voluntary at discharge (CTO => VOL), 88.9% were born in Australia. In the group who was subject to a CTO and admission and discharge (CTO = CTO), 88.3% were born in Australia. This shows that for those who were born in Australia, there was not much difference in those who were voluntary at discharge or who were subject to a CTO at discharge.
Patel et al., (2011, UK)	Community Treatment Order (CTO)	To describe CTO usage regarding patient characteristics, prescribed medication and CTO conditions.	Health administrative data from from the South London and Maudsley (SLAM) NHS Foundation Trust, London, United Kingdom.	Prospective cohort study with consecutive sampling	*n* = 195	White, Asian, Black, Mixed, Other	52.3% of patients placed on CTOs were of Black ethnic origin, a figure higher than what general population data would suggest. However, when compared to mental health service data from the same area, this overrepresentation was not statistically significant.
Segal and Burgess (2006, Australia)	Conditional Release	To examine the protective value provided by conditional release and its contribution to mortality risk among patients with severe mental disorders who required hospitalisation.	The Victorian Psychiatric Case Register (VPCR)	Retrospective cohort study	8,879 patients who experienced at least one conditional release, 16,094 who had not (*n* = 24,973)	Country of birth: Australia, English-speaking background, non–English-speaking background, Unknown	People from non-English speaking backgrounds had a greater proportion of patients ever receiving conditional release compared to those who were not conditionally released and those with a history of hospitalisation. In contrast, those born in Australia, those from English-speaking backgrounds, and those with unknown background information had similar proportions of those ever conditionally released compared to the other groups.
Segal et al. (2009, Australia)	Community Treatment Order (CTO)	To assess whether the introduction of CTOs in Western Australia reduces the length of inpatient stays by offering an alternative to prolonged hospitalisations.	Linked health administrative data from Western Australia	Retrospective cohort study	129 CTO cases, 117 matched controls (*n* = 246)	Aboriginal ancestry	Aboriginal ancestry did not significantly predict hospital stay reduction. While there was a trend toward a reduction of 18.27 days, it was not statistically significant, indicating that Aboriginal ancestry did not significantly influence the change on the duration of hospital stays in this study.
Segal et al. (2017, (Australia)	Community Treatment Order (CTO)	To examine whether psychiatric patients placed on community treatment orders (CTOs) in Victoria, Australia, have a greater need for treatment to protect their health and safety than patients not placed on CTOs. It also examined whether treatment is provided in a least restrictive manner, in a way that contributed to reduced use of psychiatric hospitalisation.	The Victorian Psychiatric Case Register/RAPID data system. These records were then linked to the records of Corrections Victoria, the Socio-Economic Indexes for Areas, Australian Mental Health Outcomes and Classification Network’s (AMHOCN’S) Health of the Nation Outcome Scales (HoNOS) records	Retrospective cohort study	11,424 placed on a CTO, 16,161 not placed on a CTO (*n* = 27, 585).	Aboriginal or Torres Strait Islander	3% of the CTO cohort were of Aboriginal or Torres Strait Islander background (which is the same percentage of people who are Aboriginal or Torres Strait Islander in the hospitalised patient sample in Victoria).
Silva et al. (2019, Switzerland)	Community Treatment Order (CTO)	To examine the incidence and prevalence of CTOs in Western Switzerland and describe the population profile, orders duration and reasons for discharge.	Public Health Service (PHS)’s data on CTOs initiated between the 1st of January 2013 and the 31st of December 2017.	Retrospective cohort study	*n* = 241, from Canton of Vaud, Switzerland	Swiss Origin	People subject to CTOs were mainly of Swiss origin (74%). Not being of Swiss origin was associated with a longer duration of being subject to a CTO.
Suetani et al. (2022, Australia)	Community Treatment Order (CTO)	To compare the demographic, clinical, social functioning, substance use and service utilisation profiles of people living with psychosis under CTOs with those who were not from five Australian states	The 2010 Survey of High Impact Psychosis (SHIP) data.	Cross-sectional survey with a two-phase design	*n* = 1,612	Aboriginal or Torres Strait Islander heritage, country of birth (born in Australia or not born in Australia), and language spoken at home (English or non-English).	When examining demographic characteristics of people under CTOs compared to people not under a CTO, there were no differences between the two groups in the following variables: country of birth, Aboriginal or Torres Strait Islander descent, language spoken at home.
Swartz et al. (2001, USA)	Outpatient Commitment (OPC)	To provide empirical data on involuntary outpatient commitment and to evaluate its effectiveness in improving outcomes among persons with severe mental illnesses.	Data from patient report, treatment record, hospital admission, and arrest record	Randomised Control Trial	*n* = 331	African American, White/Other	Approximately two-thirds (majority) were African American
Swartz et al. (2002, USA)	Outpatient Commitment (OPC)	To examine self-reported coercion in subjects with severe mental illness who were randomly assigned in an experimental study to continue under, or be released from, involuntary outpatient commitment (OPC) after hospital discharge in North Carolina, USA	Semi-structured Admission Experience Interview (AEI) and structured MAES	Randomised Control Trial	*n* = 331	African American, White/Other	Higher levels of coercion among were reported by African Americans under OPC.
Van Dorn et al. (2006, USA)	Mandated Community Treatment	To explore the link between receipt of mandated (or ‘leveraged’) community treatment and reasons for avoiding or delaying treatment reported by persons with severe mental illness, and to examine the potential moderating effect of social support on the association between mandated treatment experiences and barriers attributable to fear of involuntary commitment or forced treatment.	Survey of people with psychiatric disorders being treated in public-sector mental health service systems in five U.S. cities. Including items adapted from the Duke Mental Health Study, the anchored version of the *Brief Psychiatric Rating Scale and* a subscale of the MacArthur Admission Experiences Scale modified for outpatient use.	Cross-sectional study	*n* = 1,011	White, Non-White	People identified as white where more likely to have mandate-related barriers to care amongst patients subjected to mandated community treatment. Mandate-related barriers to care included delaying getting help for mental health, alcohol or drug problems due to feat of being forced to take medicine or treatment that they don’t want, or fear of being involuntary committed.
Weich et al. (2020, (UK)	Community Treatment Order (CTO)	To examine variation in the use of CTOs and their associations with patient outcomes and health-care costs from 61 NHS mental health provider trusts	Linked health administrative data.	Retrospective cohort study	*n* = 69,832	Ethnicity (Mixed, Asian, Black, Other) vs. White	Black patients were more likely to be on CTOs, variations were also observed based on location.
